# Evaluating machine learning classifiers for glaucoma referral decision support in primary care settings

**DOI:** 10.1038/s41598-022-12270-w

**Published:** 2022-05-20

**Authors:** Omkar G. Kaskar, Elaine Wells-Gray, David Fleischman, Landon Grace

**Affiliations:** 1 North Carolina State University, Raleigh, NC 27695 USA; 2 Lumata Health, 1111 North Lee Ave, Oklahoma City, OK 73103 USA; 3 University of North Carolina at Chapel Hill, Chapel Hill, NC 27599 USA

**Keywords:** Glaucoma, Engineering

## Abstract

Several artificial intelligence algorithms have been proposed to help diagnose glaucoma by analyzing the functional and/or structural changes in the eye. These algorithms require carefully curated datasets with access to ocular images. In the current study, we have modeled and evaluated classifiers to predict self-reported glaucoma using a single, easily obtained ocular feature (intraocular pressure (IOP)) and non-ocular features (age, gender, race, body mass index, systolic and diastolic blood pressure, and comorbidities). The classifiers were trained on publicly available data of 3015 subjects without a glaucoma diagnosis at the time of enrollment. 337 subjects subsequently self-reported a glaucoma diagnosis in a span of 1–12 years after enrollment. The classifiers were evaluated on the ability to identify these subjects by only using their features recorded at the time of enrollment. Support vector machine, logistic regression, and adaptive boosting performed similarly on the dataset with F1 scores of 0.31, 0.30, and 0.28, respectively. Logistic regression had the highest sensitivity at 60% with a specificity of 69%. Predictive classifiers using primarily non-ocular features have the potential to be used for identifying suspected glaucoma in non-eye care settings, including primary care. Further research into finding additional features that improve the performance of predictive classifiers is warranted.

## Introduction

Glaucoma is a progressive optic neuropathy resulting in the loss of retinal ganglion cells; if untreated it can result in complete blindness. It is the leading cause of irreversible blindness in the world. At present, it affects approximately 70 million people, with the number projected to grow to about 112 million by 2040^1^. Although the visual impairment caused by glaucoma is irreversible, early detection and treatment of the disease can reduce the risks of permanent vision loss^2^. Unfortunately, this is hampered by the asymptomatic nature of glaucoma^3^ and its complex, resource-intensive, and subjective diagnostic process^4–7^. Artificial intelligence (AI)-based approaches may enable the construction, validation, and implementation of predictive models to identify individuals who are at high risk of developing glaucoma, in settings that do not necessarily have access to ophthalmic imaging devices (e.g. primary care) and coordinate their care to ophthalmology.

In recent years, several AI-based approaches have been explored for the diagnosis of ophthalmic pathologies such as diabetic retinopathy^8,9^, macular edema^10,11^, and keratoconus^12^. Some of these efforts have resulted in new medical devices. In 2018, IDx-DR was approved by the US Food and Drug Association as the first fully autonomous AI-based diabetic retinopathy diagnostic system^13^. Several AI studies have attempted to interpret the structural and functional patterns manifesting in the eye for the prognosis and diagnosis of glaucoma^14–25^. Artificial neural networks (ANN) and machine learning classifiers have been used on functional data, such as visual fields, to identify patterns of glaucomatous progression earlier than more conventional methods^14–17^. The advent of deep learning has allowed the use of retinal imaging such as color fundus photographs (CFPs)^18–22^ and macular optical coherence tomography (OCT) images^23–25^ to extract structural features to differentiate glaucomatous damage. Compared to conditions such as diabetic retinopathy, where clinically feasible AI-based diagnostic technologies have already been adopted, it may be more difficult to develop such tools for glaucoma, owing to the significant variation in the appearance of the optic discs. The need for carefully chosen, large, and diverse training datasets to achieve high diagnostic accuracy adds to this challenge. The performance of glaucoma-specific models depends on the quality and number of images (> 100,000), making it a time consuming and expensive process^26^. Furthermore, repeated visual field tests are required to account for their inherent subjectivity, making them a major part of the workload being placed on hospital eye services^22,27^.

Relatively few AI-based studies have focused on developing predictive models for glaucoma without using visual fields, CFPs, or OCT images. Leveraging the wide adoption of electronic health records (EHR), Baxter et al.^28^ used machine learning classifiers to identify high risk patients with open angle glaucoma (OAG) requiring surgery within 6 months. Mehta et al.^29^ trained multiple ensemble models, each using a different set of features, to differentiate between glaucomatous and healthy eyes. One of those models used demographic, systemic, and ocular data to make these classifications. Similarly, Tielsch et al.^30^ fit logistic regression models to a population-based survey to screen for glaucoma, using demographic and other known risk factors. These prior studies have shown the potential for AI-based models to be used for managing glaucoma more effectively, making informed referrals to ophthalmologists, implementing more efficient population-based glaucoma screening, and developing intelligent self-monitoring devices. However, since the models by Baxter et al. and Mehta et al. are not trained on features from an undiagnosed population, they cannot be applied directly to predict glaucoma in the general population outside of those already actively managed by an eye care provider. On the other hand, although Tielsch et al. have trained their model on an undiagnosed population, they have reported very low predicted probabilities; likely a direct consequence of the dataset used.

In the current study, we have trained the classifiers on a sufficiently large cohort of subjects with a negative glaucoma diagnosis at the time of enrollment, such that it could be representative of the general population. Some of the subjects subsequently self-reported glaucoma. Different machine learning classifiers were evaluated on their ability to identify these subjects based on a combination of their demographic, systemic, ophthalmic, and comorbidity information taken at the time of enrollment. The goal was to explore the use of easily available data to inform referral decisions to eye care from primary care settings, without the use of expensive and/or time-consuming data such as visual fields or retinal images.

## Methods

The National Institutes of Health’s (NIH) Age-Related Eye Disease Study (AREDS) database was used to develop and evaluate the machine learning classifiers. The AREDS was a 12-year, multi-center, prospective study carried out to determine the risk factors associated with age-related macular degeneration (AMD)^31^. Anonymized natural history data of subjects was made publicly available by the NIH for research purposes. The corresponding author was granted access to the AREDS data by the National Eye Institute Data Access Committee, NIH and the analysis was conducted in agreement with the approved research use statement (data access request no. #89148–1). The AREDS was adherent to the tenets of the Declaration of Helsinki and was compliant with the Health Insurance Portability and Accountability Act^32^. For the present study, non-genetic data was used, which consisted of demographic, systemic, ocular (IOP), and co-morbidity information for enrolled subjects. Glaucoma was established through self-report, in which subjects were annually queried whether they had been diagnosed with glaucoma by an eye care provider. Subjects with glaucoma were determined based on those who selected ‘Yes’ from a predefined list of answers to the question ‘Has a doctor ever told you that you have glaucoma?’ The earliest diagnosis was recorded within a year of the start and the latest at over 12 years. While limited due to the lack of a “confirmed” diagnosis, the AREDS data provides a unique opportunity to build predictive models using data of non-glaucomatous subjects at the time of enrollment, some of whom subsequently self-reported new onset of glaucoma.

Information was extracted from the AREDS database for subjects who had multiple follow-up visits. The models were based on demographic features (age, gender, and race), systemic features (body mass index (BMI), systolic and diastolic blood pressure), a single ocular feature (IOP in the right and the left eye), and comorbidities (diabetes, arthritis, and AMD). The blood pressure readings were taken by a certified examiner using a mercury sphygmomanometer^33^. IOP for the AREDS participants was measured using an applanation tonometer or a pneumatonometer by experienced professionals^33^, which is representative of how IOP is measured during eye exams as part of primary eye care^34^. There were 7 non-glaucomatous subjects with missing entries for either IOP or BMI. Due to the relatively low number of missing values, imputation was not performed and these cases were removed from the dataset. A statistical quantitative description of the features is shown in Tables 1 and 2. The total number of subjects in the final database was 3,015, all of whom were non-glaucomatous when the information highlighted in Tables 1 and 2 was recorded. In the subsequent follow-up visits, 337 subjects self-reported to have been diagnosed with glaucoma (positive class) which left 2,678 non-glaucomatous (negative class) subjects at the end of the study period.

**Table 1:** Quantitative description of categorical features of subjects at the time of enrollment.

Features	Categories	Total (N = 3015)	Glaucoma Count (%)	Non-glaucoma Count (%)
Gender	Male	1353	167 (12.3%)	1186 (87.7%)
	Female	1662	170 (10.2%)	1492 (89.8%)
Race	White	2913	315 (10.8%)	2598 (89.2%)
	Black	84	18 (21.4%)	66 (78.6%)
	Hispanic	9	1 (11.1%)	8 (88.9%)
	Asian	4	1 (25%)	3 (75%)
	Other	5	2 (40%)	3 (60%)
Diabetes	Positive	239	32 (13.4%)	207 (86.6%)
	Negative	2776	305 (11%)	2471 (89%)
Arthritis	Positive	1354	157 (11.6%)	1197 (88.4%)
	Negative	1661	180 (10.8%)	1481 (89.2%)
AMD*	Category 1	746	90 (12.1%)	656 (87.9%)
	Category 2	673	65 (9.7%)	608 (90.3%)
	Category 3	1054	119 (11.3%)	935 (88.7%)
	Category 4	542	63 (11.6%)	479 (88.4%)

*AMD category descriptions^33^.

Category 1: A few small or no drusen.

Category 2: Many small drusen or a few medium-sized drusen in one or both eyes.

Category 3: Many medium-sized drusen or one or more large drusen in one or both eyes.

Category 4: Breakdown of light-sensitive cells and supporting tissue in the central retinal.

Area or abnormal and fragile blood vessels under the retina.

**Table 2:** Statistical summary of the numerical features of the subjects at the time of enrollment.

Feature	Glaucoma (Self-report at end of study, N = 337)				Non-glaucoma (N = 2678)			
	Mean	Standard deviation	Maximum	Minimum	Mean	Standard deviation	Maximum	Minimum
Age	70.3	5	81.6	56.3	69.4	5	81.7	55.8
Systolic blood pressure	138.6	18	200	100	137	18	220	70
Diastolic Blood pressure	79.2	9.7	120	50	78.5	9.5	120	42
BMI	27.9	4.8	45.6	18.2	27.4	4.8	58.2	8.9
IOP (right eye)	18.2	3.6	30	10	15.8	3.1	26	5
IOP (left eye)	18.3	3.7	30	10	15.9	3	30	4

Further data preprocessing was carried out in the steps highlighted below:

### Encoding categorical data

The pandas^35,36^ library in python was used for initial data processing. Ordinal encoding was used for the AMD categorical variable where the integer values (1–4 in increasing order of severity) had a natural ordered relationship. For all the other categorical variables (gender, race, presence of diabetes and arthritis), since no such ordinal relationship existed, dummy encoding was implemented using the one-hot encoder.

### Train test split

To estimate the generalization error of the classifiers, a nested cross-validation strategy was applied. Five randomly generated splits ensured that 80% of the data was used for training and the remaining 20% was used for testing each of the fitted classifiers. The train and test sets were stratified to have a similar ratio of glaucoma to non-glaucoma subjects. Grid search was performed for hyperparameter tuning by using a fivefold cross-validation on the training set. The best hyperparameters identified through the grid search process were then used to evaluate how the classifiers performed on the test set. The model metrics are reported as a mean over all the executions for evaluating and comparing the performance of each classifier.

### Class imbalance

The current dataset had 11% positive cases and 89% negative cases, making it an imbalanced dataset. This might result in biased classifiers that have poor predictive capabilities, specifically for the minority class. To address class imbalance, synthetic data was generated using the synthetic minority over-sampling technique (SMOTE)^37^. The algorithm works by generating new instances of the minority class rather than creating copies of the existing samples. Synthetic examples are introduced along the line segments joining each sample of the minority class and any/all of its nearest neighbors, determined by the Euclidean distance between them.

### Model training

The Scikit-learn^38^ and the Keras^39^ libraries in Python were used to build the classifiers. The classifiers included a linear method (logistic regression), a non-linear method (support vector machines), and an ensemble method (Adaptive boosting). The performance of different classifiers is usually evaluated using metrics such as accuracy, precision, recall, specificity, F1 score, and area under the curve (AUC) for the receiver operating characteristic (ROC) and precision-recall curves. With imbalanced data, the regular measures of performance such as accuracy are often misleading. Recall (i.e., sensitivity) measures the ability of the model to correctly identify the positive class (i.e., glaucomatous subjects). Precision (i.e., positive predictive value) indicates the proportion of correct positive predictions. Since there is usually a tradeoff between precision and recall, their harmonic mean, called the F1 score is often used. In the current study, the classifiers’ hyperparameters are optimized such that they maximize the F1 score. A grid search technique was used in the inner loop of the nested cross-validation to identify the optimized hyperparameters. A brief description of the setup for each model and the hyperparameters chosen via cross-validation are given below:

#### Logistic regression

Logistic regression is a linear model for classification that uses the logistic (sigmoid) function to estimate the probability that a sample with given features belongs to the default class (Y = 1). The probability predictions are transformed into a binary output (0 or 1) using a threshold of 0.5 in scikit-learn. The logistic regression classifier was set up using the limited-memory Broyden–Fletcher–Goldfarb–Shanno (LBFGS) solver. L2 regularization was used to prevent overfitting and the inverse regularization coefficient, *C*, was set at 0.001. The maximum number of iterations for the solver to converge was 10,000. Standardization of the dataset was carried out so that all features are approximately centered around zero and have a unit variance. This ensures that the regularization is applied equally to all the features.

#### Support vector machines

The support vector machine (SVM) classifier determines a hyperplane that directly classifies samples into one class or the other. A non-linear decision function can be determined using a kernel function that implicitly maps the features into a high-dimensional space. In the present study, a radial basis function kernel was used, and the inverse regularization coefficient, *C,* was set at 0.001. The kernel coefficient, *γ*, represents the inverse of the radius of influence of samples selected by the model as support vectors, and was set as 0.0001. Similar to the setup of the logistic regression classifier, the SVM classifier is not scale invariant and the features thus were standardized prior to training.

#### Adaptive boosting

Adaptive Boosting (AdaBoost) fits a sequence of weak learners such as decision trees, with a single internal node (decision stumps), on data that is repeatedly modified by assigning weights. At each boosting iteration, the examples that are misclassified by the boosted model at the previous step are assigned a higher weight, while the weights for the correctly classified examples are decreased. Each subsequent weak learner thereby focusses more on correctly classifying the examples that are missed by the previous ones. A weighted majority vote is taken from all the weak learners to determine the final classification. 200 decision stumps were used in the current model. The learning rate controls the contribution of the new decision stump to the existing model and is suggested to be set to small values (< 0.1)^40^. In the current model the learning rate is set as 0.01.

A permutation feature importance technique was used to determine the predictive power of the features used. The technique was set up to calculate the drop in the F1 score when a single feature value was randomly shuffled^41^. This results in breaking the pattern between the feature and the target class, and the drop in F1 score is indicative of the importance of that feature to the model. If a particular feature is important to the model, randomly shuffling its values will deteriorate the performance of the model, while doing the same to a relatively less important feature would not adversely affect the model’s performance. Permutation feature importance was preferred over the impurity-based ranking technique used in decision tree classifiers, as it is model agnostic and is unbiased towards features exhibiting high cardinality (most numerical features)^42^.

## Results

**Figure 1:**
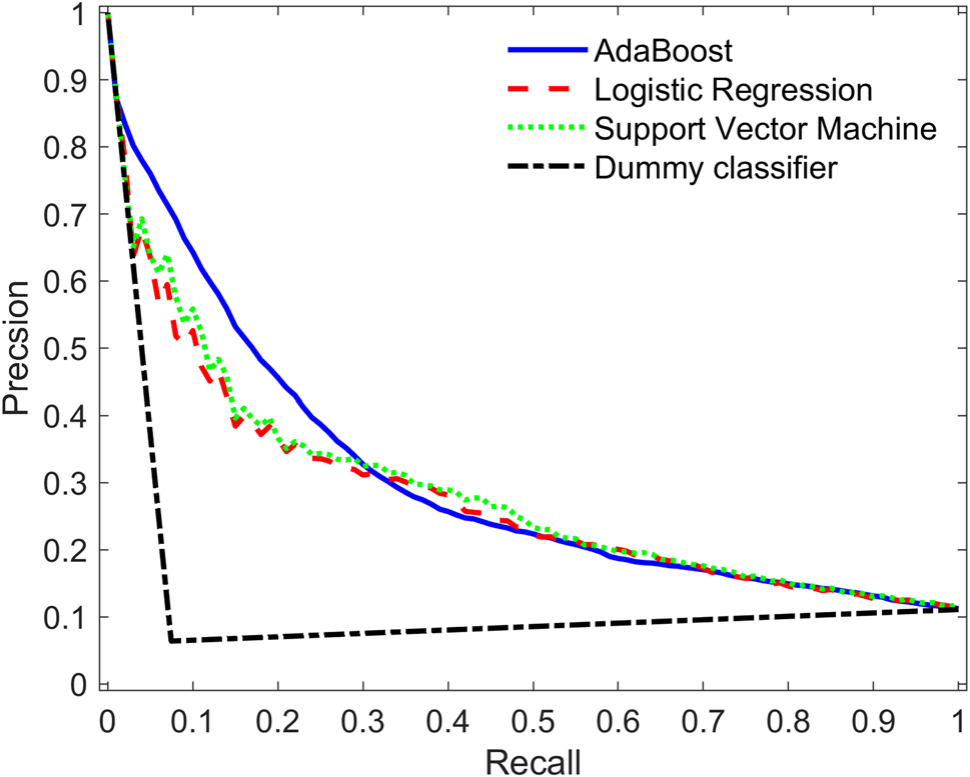
The average Precision-Recall curves for all classifiers with respect to a dummy classifier. The area under the curve (AUC) reported as mean (standard deviation): Adaptive boosting (AdaBoost) – 0.30 (0.07), support vector machine – 0.29 (0.05), and logistic regression – 0.28 (0.05).

Table 3 shows the performance of the classifiers based on the primary outcome measures: sensitivity (or recall), specificity, F1 score, accuracy, and area under the precision-recall curve. As mentioned earlier, sensitivity is a measure of the false-negatives, and F1 score is a harmonic mean of the sensitivity and precision. Specificity is a measure of the ability to correctly classify as negative (i.e., non-glaucomatous) those without the disease. As seen in Table 3, all three machine learning classifiers perform similarly. This is highlighted in Fig. 1, which shows the average precision and recall curves for all classifiers relative to one from a dummy classifier that makes random classifications. The precision-recall curve is more informative than the ROC curve when evaluating models with class imbalance^43^. The area under the precision-recall curve for all three classifiers is greater than the dummy classifier: 0.30, 0.29, and 0.28 for AdaBoost, SVM, and logistic regression, respectively. Classifiers that have a greater area under the precision-recall curve compared to that of the dummy classifier are indicative of their learnt ability to identify patterns in the data.

**Table 3:** Performance metrics reported as mean (standard deviation) over all the executions.

Models (N = 25)	Sensitivity/ Recall	Specificity	F1 score	Accuracy	Area under precision-recall curve
Support Vector Machine	0.52 (0.06)	0.77 (0.03)	0.31 (0.04)	0.74 (0.03)	0.29 (0.05)
Logistic Regression	0.60 (0.07)	0.69 (0.02)	0.30 (0.03)	0.68 (0.02)	0.28 (0.05)
AdaBoost	0.57 (0.11)	0.69 (0.06)	0.28 (0.03)	0.68 (0.04)	0.30 (0.07)
IOP greater than 21 mm Hg	0.25	0.93	0.28	0.86

Traditionally, glaucoma screening programs and referrals for comprehensive eye examination have been made on the basis of IOP, with individuals having IOP > 21 mm Hg considered to be at high risk for glaucoma^30,44,45^. Table 3 also shows the performance of a similar criterion applied on the current dataset. Subjects with IOP > 21 mm Hg in either eye were predicted to have glaucoma. With the traditional IOP criterion, the sensitivity is very poor when compared to the machine learning classifiers, as reported in Table 3. Based on the sensitivity, machine learning classifiers are likely to identify more than twice as many subjects with glaucoma from the current dataset.

**Figure 2:**
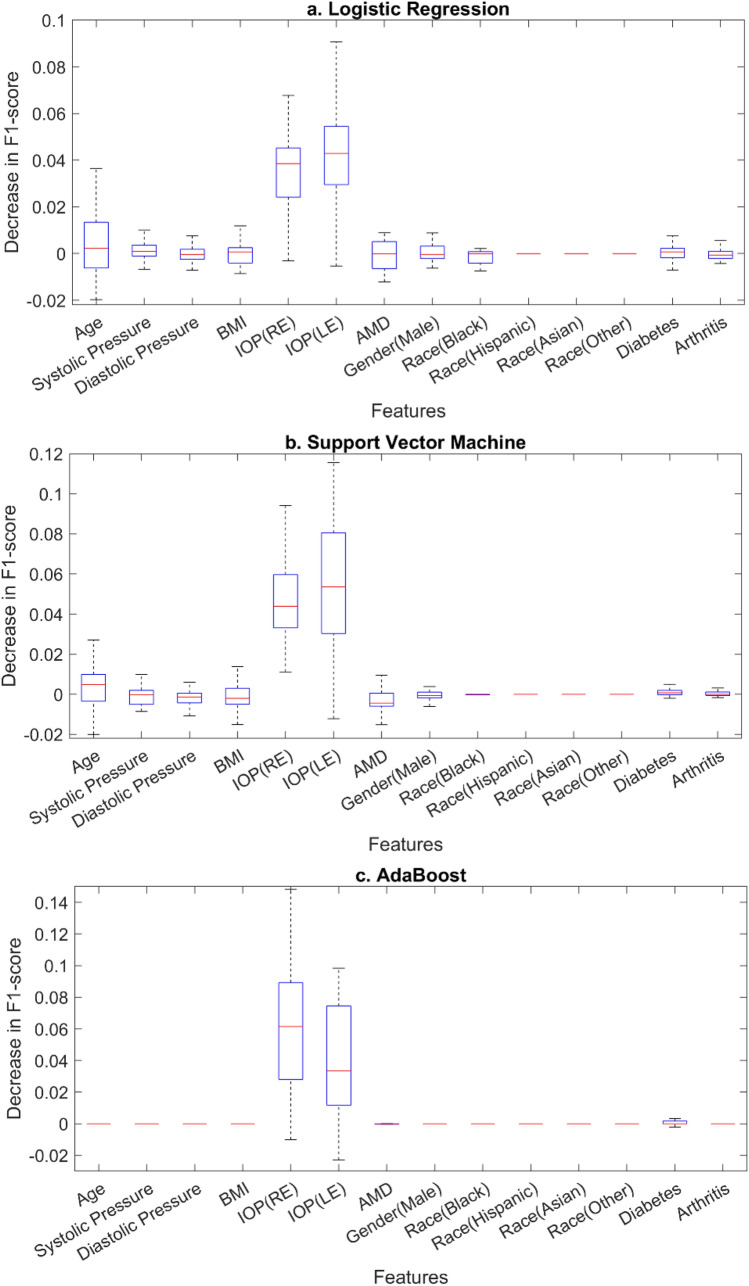
Permutation feature importance applied to each classifier: (**a**) Logistic regression, (**b**) Support vector machine, and (**c**) Adaptive boosting (AdaBoost). Mean decrease in F1 score is shown for each feature: age, systolic and diastolic blood pressure, gender (male), body mass index (BMI), intraocular pressure (IOP) in the right eye (RE) and left eye (LE), age-related macular degeneration (AMD) category, race (black, Hispanic, Asian, and other), and presence of diabetes and arthritis.

The predictive capabilities for the features used for classification were evaluated using the permutation feature importance technique. Figure 2 shows the drop in the F1 score for the 3 classifiers as each feature was permuted. The features which contribute most to the F1 score are IOP and age. The age of the patient has more predictive capabilities in case of logistic regression and support vector machine as compared to AdaBoost.

## Discussion

The classifiers were trained and evaluated on the AREDS dataset, which had subjects who were all non-glaucomatous when their baseline features were recorded. Self-reporting of glaucoma diagnosis is a limitation; it may be subject to inaccuracies due to the lack of a widely accepted method of confirming diagnosis, the associated potential for misdiagnosis from a provider, and/or misunderstanding of a diagnosis (or lack thereof) by the patient. However, self-reported glaucoma status has been regularly used in previous studies^46–48^, and its usefulness and performance has been explored^49^.

The AREDS dataset has certain advantages that address some of the sources of bias that often appear in AI-based studies^50^. First, the final database has 3,015 subjects, which is a relatively large cohort which results in more realistic performance of the classifiers. Second, unlike other studies where the data is retrieved from glaucoma clinics in which there is a higher proportion of glaucoma cases, the current study has 337 glaucomatous cases and 2,678 non-glaucomatous cases, which represents a more likely real-world scenario. Third, the prevalence of comorbidities such as diabetes, arthritis, and AMD are taken into consideration as some people would most likely present with multiple diseases. AI-based studies that use structural and functional data^14,19^, generally consider an otherwise-healthy but glaucomatous population, but patients often present with multiple conditions. This approach would likely help the classifiers generalize better to real world data. The classifiers were trained on subjects’ age, gender, race, BMI, systolic and diastolic blood pressures, IOP, and prevalence of comorbidities.

Table 4 summarizes other AI-based glaucoma risk prediction studies that do not use visual field tests, CFPs, and OCT scans, alongside the current one. The model developed by Baxter et al.^28^ predicts patients at high risk of glaucoma progression as represented by the need for surgical intervention within 6 months. The model was trained on EHR data of patients already diagnosed with glaucoma. Their final dataset was relatively small with 385 total patients, all of whom had glaucoma, and 174 of whom underwent surgery. Therefore, their model would not be appropriate to determine individuals with glaucoma from an undiagnosed population. Mehta et al.^29^ trained their model on a labeled dataset with healthy and glaucomatous eyes using demographic, systemic, and ocular information. Since there is value in predicting glaucoma in the general population, they also applied the model on a cohort of fifty-five subjects without a glaucoma diagnosis at the time of data collection, all of whom subsequently developed glaucoma. Although their model predicts glaucoma with an accuracy of 75%, a cohort of fifty-five subjects is very small to assess its predictive capabilities. Tielsch et al.^30^ reported sensitivities and specificities of their logistic regression models for various decision thresholds (0.025, 0.05, 0.1, 0.15). They noted that the range of predicted probabilities from their model were below 0.2, and the sensitivity and specificity, with 0.15 as the probability cut-off, were 35% and 97%, respectively. By contrast, in the current study we used a standard probability threshold of 0.5 for the logistic regression classifier. Their data had 191 glaucoma cases and 5,054 normal cases, making it a highly imbalanced dataset. They have not reported whether any sampling techniques were employed prior to fitting the model to address the imbalance. This might have resulted in a model that is biased toward predicting the more frequently occurring class. As seen in Table 4, the current study mitigates some of the limitations addressed above.

**Table 4:** Summary of artificial intelligence-based glaucoma risk prediction models that do not use visual fields and imaging data.

Reference	Description	Features used	Performance
Baxter et al.^28^	Predicting need for surgical intervention within 6 months for patients (N = 385) with open angle glaucoma	48 features that can be broadly categorized into vital signs, body mass index, smoking status, comorbidities, hospitalization status, medications, and lab values	Logistic regression Accuracy: 62% Sensitivity: 78% Specificity: 50%
Mehta et al.^29^	Predicting self-report of open angle glaucoma in a population (N = 1689) without a clinical diagnosis at the time of testing	Age, gender, ethnicity, body mass index, forced vital capacity, peak expiratory flow, heart rate, diastolic and systolic blood pressure, diabetes, recent nicotine and caffeine intake, intraocular pressure, corneal hysteresis, and corneal resistance factor	Extreme gradient boosting (XGBoost) Accuracy: 75%
Tielsch et al.^30^	Predicting glaucoma in a normal population (N = 5308)	Age, race, intraocular pressure, family history of glaucoma, and diabetes	Logistic regression Predicted probability threshold ≥ 0.025 Sensitivity: 86% Specificity: 66%
Current study	Predicting self-report of glaucoma in a population without a clinical diagnosis at the time of testing	Age, gender, race, BMI, systolic and diastolic blood pressures, and comorbidities	Logistic regression, support vector machine, and adaptive boosting Accuracy: 68%–74% Sensitivity: 52%–57% Specificity: 69%–77%

Applying a permutation feature importance technique to the classifiers in the current study showed that IOP and age were the most predictive features in terms of increasing the F1 score. Systemic features, comorbidities, and racial data did not contribute to the predictive capabilities of the classifiers. However, this does not imply that the information is not important in terms of its association with glaucoma. The results of the permutation feature importance are specific to the current dataset and reflect the contribution of the features to the F1 score. Several large prevalence studies have documented African ancestry as a risk-factor for glaucoma with higher levels of IOP^51–53^. The classifiers’ lack of dependency on race is likely due to the very high prevalence of white participants in the AREDS database, which is a limitation of the current study. A more balanced racial distribution within the data may have yielded different results. Vascular conditions such as blood pressure have been investigated as possible risk-factors for glaucoma. However, results have been inconclusive. While the Egna-Neumarkt Study^54^ found an association between glaucoma and systemic hypertension, the Rotterdam study^55^ found that blood pressure was associated with high-tension glaucoma but not with normal-tension glaucoma. On the other hand, the Beijing Eye Study^56^ found that neither the systolic nor the diastolic blood pressures were significantly associated with the prevalence of glaucoma. To truly assess the importance of each feature, the dataset must be highly standardized and balanced across the different features. Additionally, majority of the AREDS participants had AMD, which is not representative of the normal population, and is a limitation of the current approach. While the feature permutation importance technique suggests that AMD may not be an important predictor for glaucoma, stratifying the dataset according to AMD categories may be a suitable approach to extract conclusive information. However, this was not pursued in the current study as it would reduce the number of samples in the dataset, making it prone to overfitting.

The aim of screening is to detect diseases early and treat conditions that have already produced pathological change but have not reached a stage where medical intervention is sought spontaneously^57^. Unlike diagnostic tools that require high sensitivity and specificity, screening can be relatively less accurate as it does not form the basis for treatment. Individuals identified through a positive screen test must be referred for diagnosis and necessary treatment. The asymptomatic nature of glaucoma results in 50%-90% undetected cases until advanced stages of the disease^4–6^ . The lack of regular visits to an ophthalmologist is one of the major causes of undiagnosed glaucoma^58^. Although these numbers support the need for glaucoma screening programs, they are not very common due to their high costs^59^ and the lack of an ideal screening method^60,61^. The Student Sight Savers Program implemented glaucoma screening for over 41,000 people in the United States^44^. The screening included a questionnaire to determine family history of glaucoma, IOP measurement, and visual function assessment. Sensitivity and specificity values for a positive screening in the individual tests were 48.6% and 68.6% for a confirmed family history of glaucoma, 22.1% and 78.6% for IOP greater than 21 mm Hg, and 58.1% and 98.6% for three or more abnormal locations on the visual field. As shown in the current study, a multivariable decision function learned through data-based techniques may provide better outcomes as compared to a fixed criterion for screening. Screening techniques must be cost-effective, simple, delivered rapidly, and should cause minimal discomfort to the subject^57^. With advances in machine learning and the relative simplicity of IOP measurement, there is a potential to address challenges that are specific to glaucoma screening and strongly support the necessity of further research into these technologies^62^.

In the future, glaucoma-specific AI-based tools will become available to clinicians for improved disease management, including the possibility of standalone or EHR-integrated referral decision support tools for primary care physicians and/or care management service providers. With the expanding power of computational resources, well curated datasets of better quality will likely make these tools highly accurate for screening and, potentially, diagnosis. The results presented here highlight the potential of these tools to play a future role in the early detection of glaucoma. These types of predictive models may make screening programs, referral decisions, and self-monitoring more efficient and effective, thereby increasing the chances of managing glaucoma more effectively, reducing the risk of vision loss, and improving quality of life.

## Conclusion

In this study we evaluated multiple machine learning classifiers on their ability to predict future self-reported glaucoma based on data that can be obtained independent of an eye care provider. The goal was to explore the potential for combining readily accessible patient data with simple IOP measurement in a non-eye care setting to inform referral decisions and, thus, increase the number of glaucoma suspects evaluated early by an ophthalmologist. The three classifiers: logistic regression, support vector machine, and adaptive boosting were trained on data recorded when no subjects reported a glaucoma diagnosis. The classifiers were able to predict subjects who subsequently reported a confirmed glaucoma diagnosis, with sensitivities ranging from 52%–60% and specificities from 69%–77%. Further research into identifying more features to improve the predictive performance of such classifiers is necessary. We envision the use of such algorithms in developing tools to be used in primary care settings for advising patients to be evaluated by an eye care provider. We believe that such a tool would add value to the clinical care of patients at risk of glaucoma who might not otherwise visit an eye care provider without a referral and encouragement from their primary care provider.
